# Plastisphere community assemblage of aquatic environment: plastic-microbe interaction, role in degradation and characterization technologies

**DOI:** 10.1186/s40793-022-00430-4

**Published:** 2022-06-24

**Authors:** Sujata Dey, Ajaya Kumar Rout, Bijay Kumar Behera, Koushik Ghosh

**Affiliations:** 1 Aquatic Environmental Biotechnology and Nanotechnology Division, ICAR-Central Inland Fisheries Research Institute, Barrackpore, Kolkata, West Bengal 700120 India; 2 Aquaculture Laboratory, Department of Zoology, The University of Burdwan, Golapbag, Burdwan, West Bengal 713104 India

**Keywords:** Plastisphere, Biofilm assemblage, Plastic-microbe interaction, Plastic degrading microbes, Plastisphere characterization

## Abstract

It is undeniable that plastics are ubiquitous and a threat to global ecosystems. Plastic waste is transformed into microplastics (MPs) through physical and chemical disruption processes within the aquatic environment. MPs are detected in almost every environment due to their worldwide transportability through ocean currents or wind, which allows them to reach even the most remote regions of our planet. MPs colonized by biofilm-forming microbial communities are known as the ‘‘plastisphere”. The revelation that this unique substrate can aid microbial dispersal has piqued interest in the ground of microbial ecology. MPs have synergetic effects on the development, transportation, persistence, and ecology of microorganisms. This review summarizes the studies of plastisphere in recent years and the microbial community assemblage (viz*.* autotrophs, heterotrophs, predators, and pathogens). We also discussed plastic-microbe interactions and the potential sources of plastic degrading microorganisms. Finally, it also focuses on current technologies used to characterize those microbial inhabitants and recommendations for further research.

**Supplementary Information:**

The online version contains supplementary material available at 10.1186/s40793-022-00430-4.

## Introduction

Plastic products are being manufactured at an enormous scale globally as they have become an integral part of daily life. Its market range consists of packaging and wrapping materials, carry bags, building materials, and industrial products. The global estimation of plastic waste generation is about 6.3 billion tons in the past few years [1, 2]. Studies have estimated that the current rate of plastic waste generation could double by 2030 if increased at this rate without any intervention [3]. Indian Plastic Waste Management Amendment Rules, 2021, has defined single-use plastics (SUP) as “a plastic commodity intended to be used once for the same purpose before being disposed of or recycled”. The Indian government has banned most SUPs from 1st July 2022 to mitigate environmental pollution. The European Commission has decided to ban some SUPs, which came into effect on 3rd July 2021. Landfill treatment of plastic wastes results in secondary environmental pollution [4–6]. Most plastic wastes (70–80%) are transported through the river to the ocean [7] and dispersed along the coastline, surface water, seafloor, and remote areas far from land [8, 9]. As per recent reports, an estimated 51 trillion plastic fragments of 236,000 tons are present in the marine environment [10]. Lebreton et al. [11] reported that about 91% of mismanaged plastic wastes are transported via watersheds (> 100 km^2^). Furthermore, > 25% of them have been discarded globally into 14 large (> 1,000,000 km^2^) riverine watersheds, viz. in Asia (Ganges, Yangtze, and Amur), Africa (Nile, Zambezi, and Congo), North America (Mississippi and Saint Lawrence), Europe (Lena and Volga), South America (Amazon). Recent multilevel dataset of microplastic abundance confirmed the presence of 8.2 × 104 ~ 57.8 × 104 tons (24.4 trillion pieces) of microplastics in the world’s upper oceans [12]. Global scenario of microplastic concentration on major aquatic bodies is mentioned in (Additional file 1: Table S1). Frias et al. [13] defined Microplastics (MPs) as ‘‘Any synthetic polymeric matrix or solid particle, with regular or irregular shape and with size ranging from 1 μm to 5 mm, of either primary or secondary manufacturing origin, which are insoluble in water”. Primary MP fragments are produced in microscopic dimensions [14]. Each year, a projected 1.5 million tons of MPs are released into the ocean [15]. Smaller MP fragments are comparatively more detrimental and difficult to eliminate than larger fragments of similar weight [16, 17]. The MPs formed from the decomposition and weathering of plastic waste in the aquatic environment converted to secondary MPs [14, 18]. The distribution of plastic waste in aquatic bodies is determined by buoyancy. Wind and ocean current facilitates the long distance transportation of floating plastics (for e.g., PE and PP) as they are less dense than seawater [19]. MPs like PVC, on the other hand, are more likely to sink due to greater density [20]. Floating plastics may lose surface hydrophobicity and eventually settle at the seafloor due to an increase in density over time [21]. They are capable of persisting for hundreds to thousands of years due to durability and stability. MPs in the form of fragments, pellets, fibre, foams, and films are most frequently collected from surface water samples [22]. MPs have a larger surface area to volume ratio and provide easier attachment for hydrophobic organic matters [23, 24].

Microplastics' physical attributes include their form, size, colour, density, and crystallinity, which are typically measured using microscopes. These characteristics can have a big impact on how microplastics behave in the environment and how harmful they are to creatures [25]. The size of microplastics is crucial to research since it is the most important property that distinguishes microplastics from conventional pollutants [26]. Microplastic density varies based on the polymers used and the manufacturing technique. Polyethylene (PE), polypropylene (PP), and polyvinylchloride (PVC), for example, have densities of 0.92–0.97 g/cm^3^, 0.85–0.94 g/cm^3^, and 1.38 g/cm^3^, respectively [27]. Lower density microplastics float on the top or suspend in water, where they can be consumed by creatures dwelling in the middle and upper levels [28]. They can also be identified by their colour, which can be utilized to determine their likely origin sources. Translucent, pink, yellow, red, green, blue, brown, black, grey, purple, and white are all prevalent colours for microplastics. Colourful samples are easier to spot in environmental media, whereas dull samples are often overlooked. The hue can also influence how long they stay in the environment and how quickly they degrade. The exposure time of the sample in the environment is represented by different degrees of fading [29, 30]. With increased exposure duration, the likelihood of oxidation and the degree of weathering increases [31]. According to certain studies, the shape of microplastics has an impact on desorption, adsorption, and ecological impacts. Sharp edges suggest a recent incursion into the ecosystem, but smooth edges indicate a long residence time, as numerous environmental pressures can produce relatively smooth edges. Another essential polymer characteristic is crystallinity, i.e., the mass ratio or volume ratio of the crystalline region [32]. The crystallinity of microplastics varies with age due to either breakdown or molecular rearrangement of polymer chains [33, 34]. The chemical composition and surface groups of microplastics are the most important chemical features. Polymers, dyes, additives (plasticizers, antioxidants), and contaminants are all adsorbed on the surface of microplastics. During the production, use, and weathering of plastics, these compounds are easily discharged into the environment [35, 36]. The physical features of polymers, such as porosity, molecular size, and degree of degradation, have an impact on the leaching rate of a chemical component [37]. Additive leakage can be facilitated by surface aging. The effect is obvious, and it is determined by the chemical content and distribution coefficient in the source plastic [38].

Studies have suggested that the adsorption of MPs in the ocean is much higher than that of sediments [39, 40]. MPs act as a unique substrate for microbial attachment due to the presence of organic compounds coupled with the various substances. The abundance of organic matter along with the coarseness of the MPs also offers a habitat for microorganisms to overcome environmental obstacles [41]. These properties make MPs an ideal substrate for microorganisms in the environment [42–44]. Plastic debris offers a surface for limiting nutrients like nitrogen, iron, and phosphorus depleting the source of nutrients in the ocean [45]. Floating MPs are a potential transport medium for microbes and even some pathogens, hastening the spread of infectious diseases [46]. The microbial colonizers on the MP surface (biofilm), known as plastisphere [47], are drifted and dispersed by currents and waves to a new habitat [46]. The identified plastisphere biomass contributes to 0.01–0.2% (approximate) of the total microbial biomass on the open ocean surface [45]. The plastisphere biomass in the ocean is probably substantial as recent studies have confirmed that only about 1% of the plastic debris that is released into the marine environment can be estimated [10]. The aquatic ecosystem and food chain are also impacted by MPs. In the ocean, MPs reportedly affected the growth and photosynthesis rate of phytoplankton [48], swimming potential of zooplankton, and hamper their reproductive efficiency [49, 50]. Researchers have also reported substantial quantities of MPs in the sediments and sea surface of the Arctic Ocean, which is supposed to be less affected by anthropogenic activities [51, 52]. MPs are reported in different levels of food web, from fish, shellfish, and coral to Antarctic krill [53–56]. MPs entering biological systems may cause local inflammation, weight loss and interfere with energy redistribution [57, 58].

In addition to it, the organic pollutants adsorbed on MP surface, such as diazinon, phenanthrene, and nonylphenol also cause ecological damage [59]. Studies have revealed that MPs also adsorb and accumulate pollutants, such as polychlorinated biphenyls (PCBs), alkylphenols bisphenol A (BPA), polybrominated diphenyl ethers (PBDEs), and dichloro-diphenyl-trichloroethane (DDTs), from the surrounding aquatic environment [2, 60, 61]. Different types of MPs with equal surface area and volume exhibits variable adsorption capacity [62]. PEs are capable of adsorbing more organic pollutants [63]. Antibiotics are also adsorbed by MPs, which accelerates the transportation of drug-resistant microbial population [64]. Subsequently, MPs can generate potential biotic and abiotic stress on the aquatic environment [65, 66]. In this review, we provided an understanding of the plastisphere, highlighting its role in plastic degradation. Moreover, these microbes may biologically transform plastic debris into detrimental compounds [67]. This review elucidates the current understanding of the composition of the plastisphere micro-ecosystem, highlighting its role in plastic biodegradation and future perspectives.

## Methodology

A relevant literature search was conducted using the following terms, *i.e.,* “plastisphere” OR “plastisphere ecology” OR “plastic degradation” OR “plastisphere characterization” OR “microplastic” OR “microplastic characterization”. Search engines for scientific research articles like Scopus, Web of Science (WoS), and Science Direct were used for this purpose. These searches consisted of papers published up to 5th January, 2022 and overall, 275 selected articles were included for the literature review. The preliminary selection method was based on the title and abstract of the paper. Those which could not be assessed by the title and abstract, were subjected to secondary quality check by scrutinizing the content of the paper. There were 65 studies on plastispheres and 62 studies on microplastics, respectively, 46 studies on plastic biodegradation, and 41 studies on various plastisphere characterization methodologies. The remaining 57 publications covered a variety of related topics such as plastic-microbe interaction, remediation procedures etc. The relevant information was collected and compiled after thoroughly reading the selected papers. There is a fair possibility that some important and relevant works might have been left uncited, specifically those that are not included in the Scopus, WoS, and Science Direct databases and did not use our article search terms.

### Plastisphere biodiversity

The term “plastisphere” was introduced by Zettler et al. [47] to describe the diverse microbial community attached to plastic surfaces and distinct from the surroundings. Environmental sampling and laboratory incubation methods are the most preferred key strategies to study the plastisphere. Although reports on benthic aquatic environments are limited due to the expense and difficulty of sampling. Studies have been executed in which plastics are incubated under artificial laboratory setups [68]. These types of experiments are usually appropriate to study the degradation capacity and enzymatic functions of microorganisms. Previous studies of the plastisphere used scanning electron microscopy (SEM) for morphological identification of different species [3]. In contrast, recent studies to investigate plastisphere mostly relied on high-through output sequencing [47]. For example, studies of marine plastisphere used the eukaryotic 18S rRNA gene and the small ribosomal subunit 16S gene (16S rRNA) for Metabarcoding with sequencing technologies such as 454 pyrosequencing and MiSeq Illumina sequencing [69]. The classification of fungal community in plastisphere is performed using Internal Transcribed Spacer (ITS) technique [70, 71]. The hypervariable 16S rRNA locus V3 and V4-V5 locus of prokaryotes are the most commonly used genetic barcodes in metabarcoding studies (Table 1). 18S rRNA is a distinctive target for the identification of microbial eukaryotes in the plastisphere. Fungal-specific primers targeting the Internal Transcribed Spacer (ITS) regions are used for the detection of fungi (Table 2). However, more future studies on the plastisphere are required.

**Table 1:** Plastisphere associated bacterial species

Bacterial species	Type of plastic	Study area	Method	References
Bacteroidetes, Proteobacteria, Cyanobacteria, Acidobacteria, and Actinobacteria	PP, PET, PE	South China Sea; surface	V4-V5 16S rRNA sequencing	[72]
Gammaproteobacteria, Actinobacteria, Opitutae, Alphaproteobacteria, and Sphingobacteria	PE, PP	Baltic, Sargasso and Mediterranean seas; surface	V3-V4 16S rRNA sequencing	[73]
Thalassospiraceae, Alteromonadaceae, and Vibrionaceae	PET	Porthcawl beach	V4-V5 16S rRNA sequencing	[74]
Flavobacteriaceae and Rhodobacteriaceae	PVC	Atlantic Ocean, Indian Ocean, Mediterranean Sea, and; seafloor	V4-V5 16S rRNA sequencing	[75]
*Pseudomonas* and *Bdellovibrio*	PS	Seawater	V4 16S rRNA sequencing	[76]
*Marinobacter*, *Idiomarina*, *Halomonas*, *Exiguobacterium*, and *Ochrobactrum*	PET, PE	Huiquan Bay (Qingdao, China); surface	V4-V5 16S rRNA sequencing	[77]
Methylologellaceae, Micrococcaceae, Pseudomonadaceae, Colwelliaceae, Haliangiaceae, Halieceaea	PE	North Atlantic	V4 16S rRNA sequencing	[78]
Flavobacteriales, Cytophagales, Rhodobacterales, Rickettsiales, Chitinophagales, Alteromonadales, and Oceanospirillales	PE, PP, PE	Mediterranean Sea; surface	V4–V5 16S rRNA sequencing	[79]
Proteobacteria and Bacteriodes	PE, PUF, PVC, PLA	York River estuary	V4–V5 16S rRNA sequencing	[68]
Saprospirae, Flavobacteriia, and Cytophagia	PETE, HDPE, PVC, LDPE, PP, PS	Coast of Bocas del Toro; sea surface	V4–V5 16S rRNA sequencing	[80]
Bacteroidetes, Firmicutes, Proteobacteria, and Cyanobacteria	–	Hikine Island, Japan	V4–V5 16S rRNA sequencing	[81]
Proteobacteria, Bacteroidetes and Cyanobacteria	–	Herzliya marina; surface	full 16S rRNA sequencing	[70]
Alphaproteobacteria, Gammaproteobacteria and Bacteroidia	PE	Offshore of Yantai, China	V4 16S rRNA sequencing	[82]
*Alcanivorax*, *Marinobacter* and *Arenibacter* genera	–	Mediterranean Sea; surface and sediment	V3-V4 16S rRNA	[83]
Actinobacteria, Cyanobacteria and Proteobacteria	–	East China Sea; deep water	V5–V6 16S rRNA sequencing	[84]
Erythrobacteraceae, Cyanobacteria, and Rhodobacteraceae	PE, PP, PS	East China Sea; surface	V3-V4 16S rRNA sequencing	[85]
*Planctomyces*, *Pirellula*, *Pseudomonas*, *Ilumatobacter*, *Synechococcus*, and *Blastopirellula*	PE, PET	Arabian Sea; surface	V4 16S rRNA sequencing	[86]
*Planctomycetacia*, *Proteobacteria*, *Nitrospira*, *Caldilineae*, and *Acidimicrobiia*	PE, PP, PS, PET, PLA	North Sea, Germany; surface	V3-V4 16S rRNA sequencing	[87]
Alphaproteobacteria, Cyanobacteria, Flavobacteria and Gammaproteobacteria	–	Mediterranean Sea; surface	V3-V5 16S rRNA sequencing	[88]
Alphaproteobacteria, Cyanobacteria, Flavobacteria, and Actinobacteria	PE	Mediterranean Sea; surface	V3-V5 16S rRNA sequencing	[89]
Rhodobacterales, Streptomycetales, Rhizobiales, and Cyanobacteria		North Atlantic subtropical gyre; seafloor	V4 16S rRNA sequencing	[90]
Alphaproteobacteria and Gammaproteobacteria	PE	North Sea; seafloor	V3–V4 16S rRNA	[71]
Cryomorphaceae, Flavobacteriaceae, Saprospiraceae	PET	North Sea; surface	V4 16S rRNA sequencing	[91]
Bryozoa, Alphaproteobacteria, Cyanobacteria, and Bacteroidetes	–	North Pacific subtropical Gyre; surface	Metagenomic sequencing	[92]

**Table 2:** Plastisphere associated fungal and phototroph species

Fungal species	Type of plastic	Study area	Method	References
*Achnanathes*, *Amphora*, *Navicula*, *Nitzschia*, *Aneumastus*, *Rhaphoneis*, *Cylindrotheca*, and *Ochrophyta*	PE	Herzliya marina; surface	18S rRNA and tufA	[70]
*Pleosporales*	PE	Herzliya marina; surface	ITS	[70]
*Cladosporium*, *Aspergillus*, and *Wallemia*	PE, PA, PU, PP, PS	western South Atlantic and Antarctic Peninsula; surface	V9, V4 18S rRNA and ITS2	[93]
Diatoms, Dinoflagellates, red, green, and brown algae	PETE, HDPE, PVC, LDPE, PP, PS	Coast of Bocas del Toro; surface	V4 18S rRNA sequencing	[80]
*Rhizidiomyces*, *Chytridium*, and *Pythium*	PE, PS	Baltic Sea; surface	V4 18S rRNA sequencing	[94]
Haptophyta, Cryptophyceae, and Chloroplastida	PE, PS	Baltic Sea; surface	V4 18S rRNA sequencing	[94]
Archaeplastida	–	East China Sea; deepwater	V4 18S rRNA sequencing	[84]
Chytridiomycetes	PE, PP, PS, PET, PLA	North Sea, Germany	V4 18S rRNA sequencing	[87]
Cryptomycota, Chytridiomycota, and Ascomycota	PE, PS	Baltic Sea; surface	V4 18S rRNA sequencing	[95]
*Paramoeba aestuarina*, *Pleurobrachia pileus*, *Paramoeba permaquidensis*, *Sagartia elegans*, *Sugiura chengshanense*, and *Rhizostoma pulmo*	PE	Belgian part of the North Sea; seafloor	ITS2	[71]
Bacillariophytina, Coscinodiscophytina	PET	North Sea; surface	V9 18S rRNA sequencing	[91]

### Plastisphere community assemblage

**Fig. 1:**
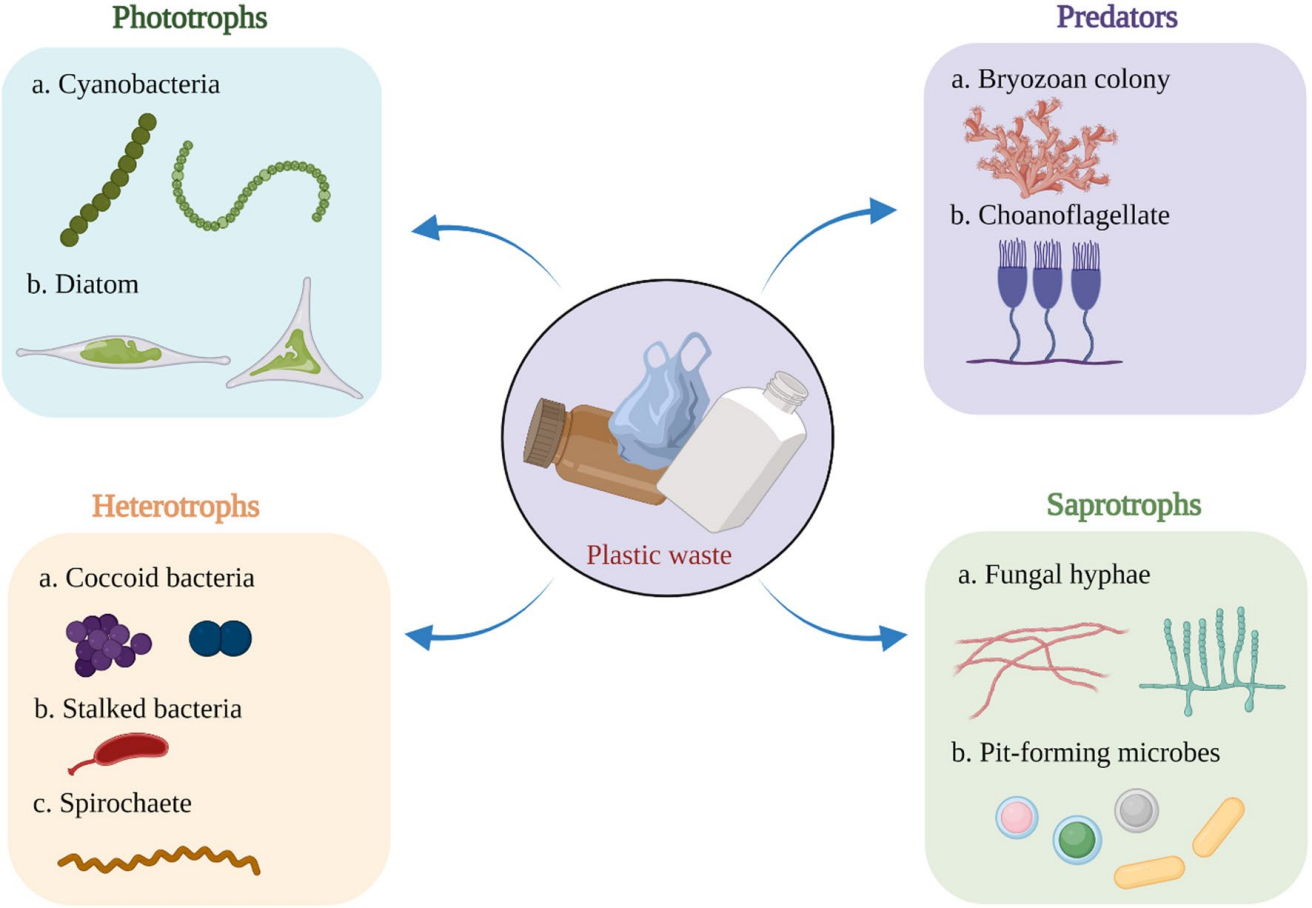
Conceptual illustration of the diverse plastisphere community, presenting a microbial ecosystem inhabiting plastic debris. Community members include phototrophs, heterotrophs, predators, and pathogens. Hypothesis drawn by a metagenomic study [92] on metabolic potential of plastisphere residents concludes that microorganisms found on plastic debris possess discrete sets of genes compared to those in a surrounding aquatic environment. The metabolic capacity and functional diversity of the plastisphere microbial community are not properly explained. Metagenome rRNA gene reads between 40 and 99% obtained from plastic debris mapped to eukaryotic rRNA, though it is unclear if the represented data is the actual abundances as eukaryotic microbial rRNA genes may consist of a significant number of disparate copies [133]

The community diversity of microbes within the plastisphere has been reported in early scanning electron micrographs of biofilms formed on plastics [96]. The term “Biofilm” can be defined as aggregates of cells that are either attached or unattached to a substrate and grow within a matrix consisting of extracellular polymeric substances (EPS) [97]. The process of biofilm formation employs a specific set of genes that are involved in the expression of adhesion, chemotaxis, communication, and substrate transport, enabling the formation of matrix and fluid channels to distribute nutrients between cells [98]. Current SEM studies [47, 87, 88, 92] combined with molecular data established the plastisphere as a surface-based micro-ecosystem (Fig. 1) comprising primary producers (phototrophs), symbionts, predators, and decomposers.

#### Phototrophs

Several studies on plastics exposed to sunlight have represented phototrophs (e.g., diatoms) as ubiquitous members of the plastisphere. They are often proclaimed as primary and occasionally dominant colonizers of plastic debris [94, 99–103]. Association between primary producers and microbial community plays significant role in aquatic food webs. Interaction between phototrophs and other microorganisms is also evident in the plastisphere [104]. Studies conducted on plastics recovered from the Sargasso Sea reported morphology [105] and amplicon reads [47] of several diatom genera including *Pleurosigma* sp., *Cyclotella meneghiniana*, *Mastogloia angulata*, *Mastogloia hulburti*, *Mastogloia pusilla* [105], *Amphora sp.*, *Nitzschia sp.* and *Sellaphora sp.*[47]. Furthermore, the genera *Amphora*, *Mastogloia*, and *Nitzschia* were also reported from the Arabian Gulf [86]. These findings imply the tendency of diatom species to inhabit the sunlit plastisphere zone. Now it has been possible to unveil the phototroph genera present in the plastisphere with the help of new chloroplast databases [106] that assigns eukaryotic phototroph data derived from amplicon studies. The collected microplastics from the Pacific and Atlantic gyres along with the data gathered from marine incubation studies validated the presence of diatoms and chlorarachniophytes (protistan phototrophs) within the ocean plastisphere [107, 108]. Amplicon sequencing and microscopic observations characterized diatoms as common members of the plastisphere, whereas metagenomic analysis [92] showed the presence of diatom clades, hinting at their replacement over the time of community maturation. Cyanobacteria are also a representative of the photosynthetic community of plastisphere [92], which is evident from the combined measurement of chlorophyll, respiration measurement, and oxygen production. Several genera of filamentous cyanobacteria, such as *Phormidium*, *Rivularia*, and *Leptolyngbya* have been found on ocean microplastics. Complementary chromatic adaptation (light-harvesting adaptation strategies) of filamentous cyanobacteria makes them capable of overcoming the high and low-light challenges of the oligotrophic open ocean [92]. A PET bottle (polyethylene terephthalate) incubation study conducted in marine waters of Oman [86] reported that 4% of the plastisphere community was occupied by *Microcystis* (cyanobacteria) indicating a possibility of transportation of harmful algal bloomers to marine waters through the propagules attached to the plastic surface. As microcystin producers like *Microcystis* are not typical inhabitants of the marine environment, this finding indicates that these colonies might have arisen from plastic sources. It was reported that plastisphere colonizing cyanobacteria exhibit an entirely different light-harvesting mechanism with a higher expression of phycobilisome antenna encoding genes [92]. This suggests that cyanobacteria of plastisphere photosynthesize through the phycobilisome complexes. Phycobilisome proteins are converted to scarcity of nitrogen, and can rapidly restructure once the nitrogen source is accessible [109]. Phycobilisome serves as a light-harvesting complex and nitrogen reservoir for cyanobacteria present in nitrogen scarce environments. High expression of nifD, nifK, and nifH (nitrogen fixing enzymes) was also reported from plastisphere [92].

#### Photoheterotrophs and heterotrophs

Photoheterotrophic genera *Erythrobacter* and *Roseobacter* are commonly present in sunlit zones of the ocean. The bacteriochlorophyll within some of them is capable of fixing CO_2_ and photosynthesizing without producing oxygen engaging in heterotrophy [110]. Mixotrophic *Roseobacter* is a less frequently encountered bacteria species [111, 112]. Several previous studies support the concept of heterotrophic utilization of organic substrates by Cyanobacteria [113, 114]. Functional genes (e.g., genes related to mixotrophy) should be taken into consideration when drawing a conclusion rather than comparison based on taxonomic genera represented on plastic debris [89, 90]. An assortment of bacterial isolates derived from cultures containing polyethylene terephthalate (PET) or polypropylene as a sole carbon source, included Firmicutes (e.g., *Bacillus spp.*) [115], Actinobacteria (e.g., *Rhodococcus spp.*) [116] and Gammaproteobacteria (e.g., *Azotobacter spp.* and *Pseudomonas spp.*) [117, 118]. Some studies [47, 90, 94, 95] precisely addressed fungal sequences growing on plastic debris. The fungal community is commonly associated with decomposition, symbiosis, parasitism, pathogenesis, and predation, playing the role of potential saprotrophs in the plastisphere. Although fungal community diversity in the plastisphere is a comparatively underexplored area, recent reports [94] on fungal assemblages, Ascomycota, Cryptomycota, and Chytridiomycota were dominant members present on polyethylene and polystyrene substrates in fresh and brackish waters, making up to 4% of the total eukaryotic reads.

#### Predators

SEM and molecular data unveiled a striking symbiotic relationship between *Ephelota* (a predatory ciliate) and its ectosymbiotic sulfide oxidizing bacteria in the plastisphere [47, 94]. Plastic debris provides the surface attachment space to these suctorian ciliates, which are also common epibionts on copepods. There is more evidence of this type of co-occurrence network [94] that has shown positive associations between *Amoebophrya* and Suessiaceae on polyethylene surfaces. In addition, *Amoebophrya* is also a typical parasite found on dinoflagellates. The predatory ciliate *Ephelota* is commonly found in microplastic samples recovered from freshwater, marine, and brackish water, which may be an important perception of this particular ciliate’s survival potential far away from the coast. A study showed the prevalence of these genera on plastic concerned with seaweed mariculture [119]. Choanoflagellates, Radiolaria, and *Micromonas* (small flagellates) also belong to the predator community in the plastisphere, which consume bacteria and other organisms.

#### Pathogens

There are reported threats from toxic chemicals and invasive microbial species associated with plastic [120]. Colonization of plastics by pathogenic microbial genera has recently been a topic of concern since the study has shown *Vibrio* and many other potential microbial pathogens can get attached to the surface of plastic debris [47]. Plastic samples recovered from tropical [121] and temperate [85, 91, 122–124], marine, as well as freshwater [125, 126] environments, reported incidence of various potential pathogens (e.g., *Arcobacter spp.*, *Aeromonas salmonicida*, and members of the group Campylobacteraceae). Microbial infections in fish, mollusks, and crustaceans [127] cause immense damage to aquaculture practices. One of the most common pathogens of fish is *Vibrio* spp., which is also responsible for the emergence of antimicrobial resistance [128]. Many of the equipment and gear used in aquaculture facilities (e.g., pens, lines, nets, floats, etc.) are plastic made which provides surface attachment area to potential harmful microbial colonizers). In addition to this, the presence of phototrophs on plastic debris which are responsible for algal blooms has also been reported [102, 129, 130]. Most of the evidence is based on molecular sequencing data and is not enough to confirm pathogenicity or toxicity, and reported abundances of pathogens were relatively low in the majority. Plastic debris can travel a long distance and frequent consumption by aquatic fauna is also evident. Aside from the risk of ingestion [120], studies have shown plastic as a potential transporter of protistan pathogens of corals [131] and fish [132]. Whenever plastic debris passes through wastewater treatment plants and into waterways, there are plenty of chances of human pathogen getting attached to its surface. A recent study [125] disclosed a relatively higher abundance of the human gastrointestinal pathogen (Campylobacteraceae) on MPs from a sewage treatment plant. Despite all the considerations, the role of plastics as means of transportation for the pathogenic microbial community is still in need of further additional investigation.

### Ecological succession

Alike any other biofilms, the formation of plastisphere usually comprises attachment, secretion of EPS (extracellular polymeric substances), and proliferation [47]. The physical properties of plastics play a key role in the selection in earlier stages of colonization, and these pioneers subsequently impact the selection of other communities within the plastisphere [134]. Microbial adhesion is affected by several environmental factors like temperature, pH, nutrient availability, and hydrodynamic conditions [135]. These variables can alter the physicochemical features of the MP surface, such as texture, hydrophobicity, and charge [136]. Microbial attachment can be accelerated in aquatic environments by increasing flow velocity, water temperature, or nutrient concentration, as long as these factors do not reach critical values [137]. Biofilm development, for example, has recently been discovered to be a social element of bacterial life. Environmental factors influence whether a cell forms or leaves a biofilm. Cells change their gene expression in response to their surroundings. Environmental influences and gene regulation are linked by second messengers like cAMP and bis-(3′-5′)-cyclic dimeric guanosine monophosphate (c-di-GMP). Cell-to-cell contact is also crucial in the formation of the biofilm. One of the most significant processes is surface attachment, which marks the transition from planktonic to biofilm mode. First, bacterial cells reversibly adhere to the surface at their poles, a process known as a reversible attachment [138, 139]. Cells undergo irreversible attachment after the intracellular second messenger, c-di-GMP, is implicated in transition [140]. Many bacteria synthesize c-di-GMP, which controls EPS synthesis and motility in opposite ways. The environmental concentration of carbon and oxygen regulates the function of these second messengers [141]. Temperature changes substantially impact biofilm shape, including thickness and cell density [142].

**Fig. 2:**
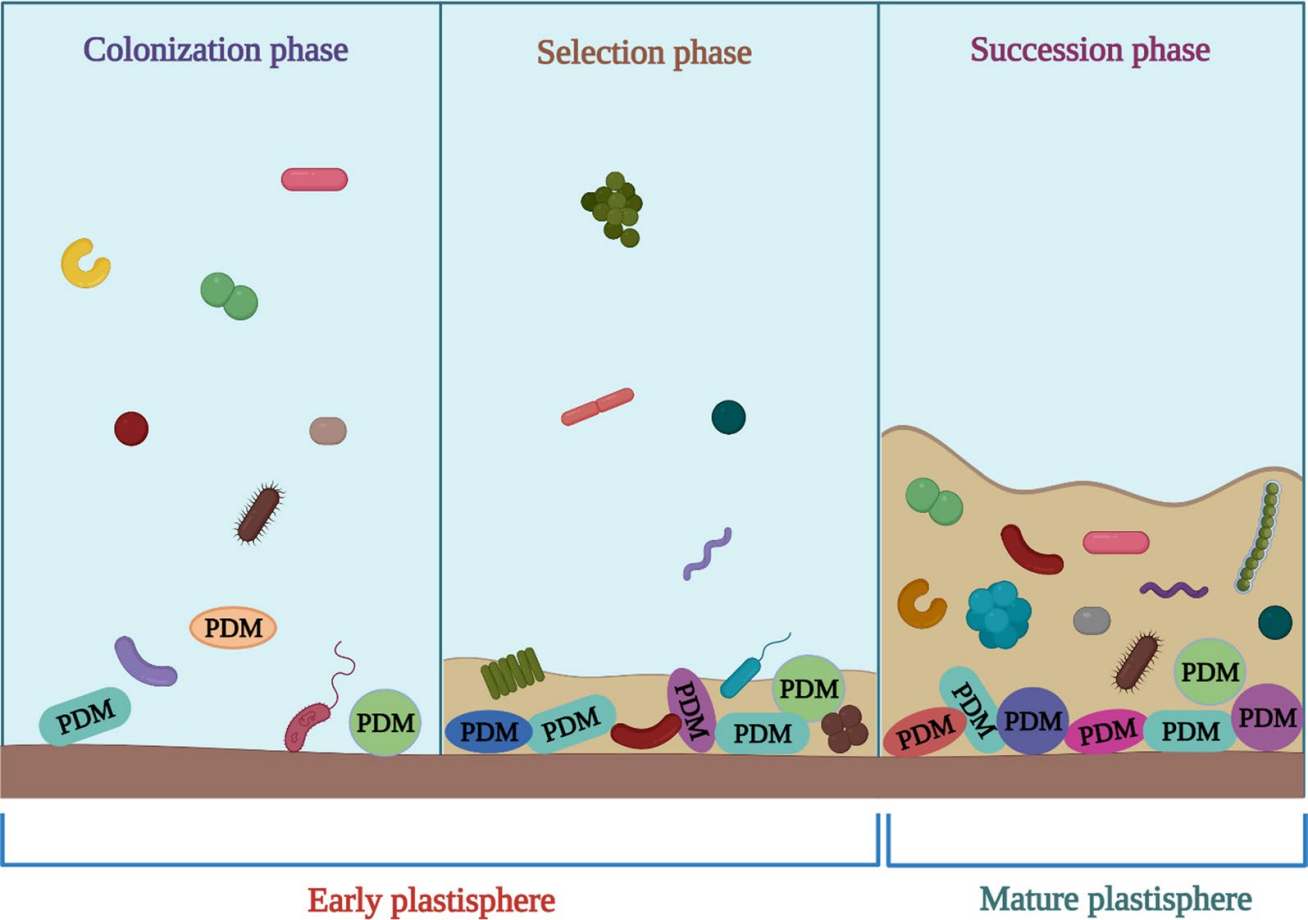
Depiction of plastic colonization in an oceanic environment in an abstract form; plastic degrading microbes (PDM) might belong to the pioneer community, especially if oligomeric and polymeric additives are available as carbon and energy sources. Once these sources are exhausted, the specialized microbes which can utilize the labile photosynthate produced by phototrophs will outcompete the PDM [158]

Studies on the plastisphere have shown strong shifts of distinct communities throughout the early stage of colonization. However, in mature biofilms, microbial communities are converged over time and remained stable. Although a consistent and stable plastisphere can be achieved within a few weeks, the formation of a mature eukaryotic community may take more time [80]. The hydrophobic property of plastics provides a strong interface to the aquatic environment obstructing the surface attachment of microbes. However, plastic surfaces are hastily covered with organic matter (collectively known as the ecocorona [66]) which facilitates microbial colonization. A preliminary study [143] has revealed that in a marine environment, the process of microbial attachment to plastics happens within minutes. The primary colonization on a pristine plastic surface is an initial proliferation of microbial colonizers devoid of high competition aiming to cover the maximum surface area within a short period. After initial colonization, microbes deviate into specific niches to avoid competing for habitat and resources. The maturing biofilm releases quorum quenching secondary metabolites as well as complex EPS (extracellular polymeric substances) for protection [123, 144–148]. At the time of colonization, readily available polymeric oligomers [149–151] or additive leachates [152], produced by weathering of plastic provide energy to pioneering microbial species. These preliminary energy sources are however exhausted within days, and a continuous supply of energy is sustained by photosynthate from other plastisphere phototrophs and organic matter obtained from the environment. As per reports [47, 92] cyanobacteria, green algae, and diatoms are pioneers of the marine plastisphere community. Studies have suggested that the plastic biodegradation process is likely to be delayed due to plastisphere community succession [153–155]. It has been reported that plastisphere colonization occurs through different succession stages: (I) Colonization or initial surface attachment of pioneering microbial community, where the copious organisms are proficient in effective surface adhesion; (II) selection phase, where polymer degrading organisms become embellished and diversified; and (III) succession phase, during which the plastic degraders are overhauled by cross-feeders, grazers and other microbes [153–156] (Fig. 2). Although microorganisms easily colonize on the MP surface, the formation of a stable biofilm takes more time [9]. Early settling members belonging to Alphaproteobacteria and Gammaproteobacteria are reported as pioneer colonizers [9]. According to reports, Gammaproteobacteria dominated in the early stages of biofilms [71, 89, 143]. *Poseobacter*, *Alteromonas*, Phodobacteriaceae, *Thalassobius*, and *Neptuniibacter* were also reported from the early stages of biofilm formation [157]. Secondary colonizers such as Flavobacteriaceae (Bacteroidetes) flourish utilizing the organic substrates released by pioneer colonizers [9, 157]. The formation of secondary biofilm requires several months [71, 158]. Tu et al. [82] reported Rhodobacteraceae (Alphaproteobacteria), Microtrichaceae (Acidimicrobiia), and Flavobacteriaceae (Bacteroidia) as the pioneers of the early biofilms (30 days). After 75 days, the biofilms are dominated by Moraxellaceae (Gammaproteobacteria) and Bacillaceae (Bacilli) groups. At the matured stage (135 days), the dominance is shifted again to the Flavobacteriaceae (Bacteroidia), with a significant increase of Microtrichaceae (Acidimicrobiia), Pirellulaceae (Planctomycetes), and Rhodobacteraceae (Alphaproteobacteria).

### Biofilm formation on aged microplastic

Physical and chemical properties of aged MPs differ from those of unaged MPs. MPs are subjected to numerous aging processes in natural environments, including ultraviolet (UV) exposure, physical corrosion, oxidation, and biodegradation [159]. Plastics in the ocean emit dissolved organic carbon (DOC), promoting heterotrophic microbial activity [150]. Aging increases the hydrophilicity, charge, and polarity of MPs [160, 161]. UV absorption by unsaturated groups, oxidation, polymer radical generation, chain cleavage, and crosslinking are all considered key processes in the aging of hydrocarbon polymers [162–164]. However, little is known about microplastics aging behavior and the effects of aging on their adsorption of contaminants in the environment. As a result, it’s critical to investigate the aging properties of microplastics concerning their age. Microplastics may coexist in natural water with reactive oxygen species (ROS) produced by natural (e.g., biological [165] and photochemical [166, 167]) and photosensitization processes [164, 168]. However, little is known about the impacts of ROS on the aging behavior of microplastics to the best of our knowledge, which could be explained by the extended times necessary for natural processes due to the low oxidant concentration in aquatic environments. Due to their porous hydrophobic architecture, MPs in the environment have been proven to be a carrier for adsorbing several sorts of heavy metals, hydrophobic organic pollutants, and pathogenic microbes. After various aging processes, MPs' surface qualities such as specific surface area (SSA) and hydrophilicity change, impacting their adsorption behavior [169, 170]. After aging, the morphology of MPs changes with increased surface roughness and specific surface area (SSA) [171, 172]. Because bacteria are prone to attach to the coarse surface of MPs, these alterations may be helpful to their adhesion on the surface [173]. UV-induced photodegradation of MPs destroys the polymer's chemical bonds, forming new functional groups [172]. They could convert PE's hydrophobic surface to hydrophilic, allowing some hydrophilic bacteria to inhabit it [174]. Aging can stimulate the release of chemicals in MPs. According to research, several chemicals can be utilized as nutrient sources to help bacteria proliferate [175]. Through hydrophobic dispersion and the formation of extracellular polymeric molecules, the biofilm boosts the adsorption capacity of contaminants in the environment [176]. Changes in weathered MPs' physicochemical properties, such as decreased surface hydrophobicity and increased heterogeneity, can, on the other hand, impact their adsorption behavior [177]. The presence of biofilms may also help MPs age more slowly due to reduced photodegradation (e.g., UV irradiation) facilitated by biofilm shields [178]. Due to a rougher surface and poorer hydrophobicity, the adsorption of nutrients, surface area for bacterial colonization, and development of biofilms are improved [47, 179].

### Plastisphere distribution

The distribution of MPs is variable in the aquatic environment, with PET, PVC, and PA dominating in the bottom sediments, while, PP and PE are mostly detected in the upper water columns [19]. This is also dependent on the surface area, volume, and density of MP fragments. The activity of the microbes is in accordance with the state of MP substrate. Reports suggest that the properties of the MPs, including roughness, surface area, hydrophobicity, surface charge, and density would be affected after biofilm formation [176, 180, 181]. Smaller MP particles are more likely to be dispersed in the deeper ocean and are relatively less studied [182]. The MPs deposited on the seafloor are transported via slower benthic currents throughout the seafloor [183]. Studies have shown that Proteobacteria and Bacteroidetes dominated the plastisphere even in deep water [89, 184]. Settling of MPs in the ocean can lead to more environmental stresses. MPs are reported to be transferred to higher organisms along the food chain [185, 186].

### Physico-chemical, microbial, and phyto remediation of plastic

**Fig. 3:**
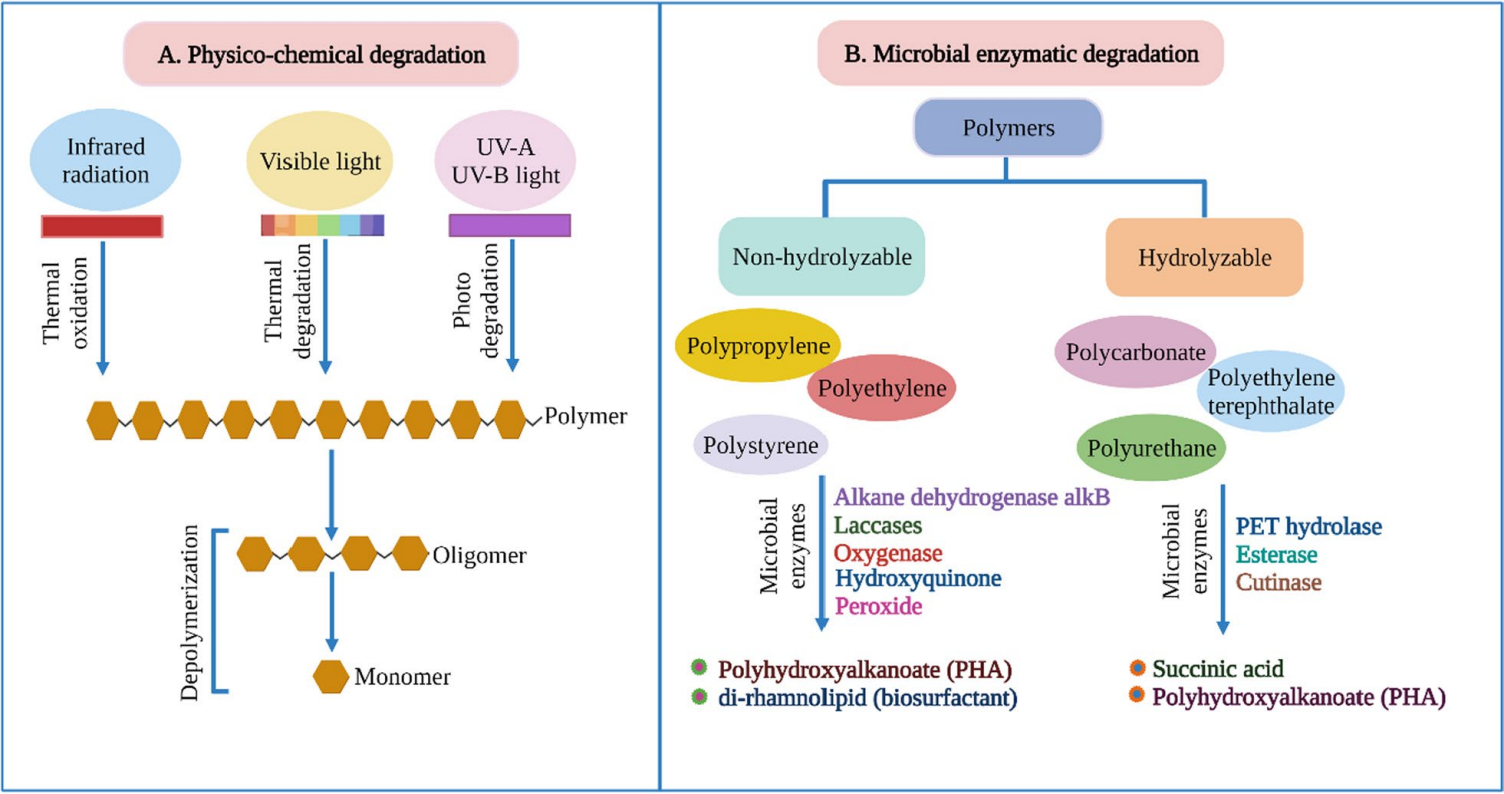
**A** Several physicochemical and biological processes interact to degrade conventional plastics. Most of the perceptions regarding biological processes are laboratory-cultured strains and consortia based, and many of these strains are present in terrestrial habitats. This diagram depicted a hypothetical representation of the processes that lead to plastic deterioration in aquatic environments like the open ocean. Floating plastic waste undergoes different types of degradation in the presence of sunlight. The visible spectrum facilitates thermal degradation, whereas the infrared radiation leads to the thermal oxidation of polymer chains, and UV is responsible for the photodegradation through the bond scission mechanism. **B** Biological pathways for polymer degradation involve the action of microorganisms growing on its surface and enzymatic processes leading to polymer hydrolysis into oligomers and eventually monomers. Hydroxyquinone, alkB, laccase, oxygenase, peroxide, etc., are reported enzymes that break down highly stable backbones of non-hydrolyzable polymers. Hydrolyzable polymers are comparatively susceptible to enzyme-mediated (PET hydrolase, esterase, cutinase, etc.) catalysis [3]

According to several studies [187–191] on previously treated polyethylene (priorly exposed to thermal treatment, UV light, or containing additives) (Additional file 1: Fig. S1) erstwhile measuring degradations, it is difficult to predict whether the microbes acted on high molecular weight polymers or its smaller fragments produced by abiotic factors (Fig. 3A). Small molecules of dissolved organic matter (low molecular weight) are leached from plastic and contribute to the microbial attachment on the plastic surface and metabolic activity in the aquatic environment. The molecular weight of the polymers must be low enough so they can pass through the microbial cell membrane and undergo intracellular oxidation [118] (Fig. 3B). Reports [192] have suggested that hydrolyzable polymers (polyethylene terephthalate, polycarbonate, polyurethane) in the photic zone act as plastisphere substrates, whereas non-hydrolyzable polymers (polypropylene, polyethylene, expanded polystyrene) frequently encountered in the pelagic zone of the marine environment (Additional file 1: Figs. S2, S3).

A group of alkane degrading enzymes such as hexadecane was reported as a potential degrader of recalcitrant polymers (polyethylene) [118]. A study [118] on mesophilic *Pseudomonas* sp. derived from marine beach sediment, incubated with a low molecular weight polyethylene (LMWPE) as a solitary carbon source has shown its potential for polyethylene biodegradation. Although hydrolyzable plastics are susceptible to microbial extracellular hydrolases (present in pre-existing degradation pathways) involved in cellulose and protein degradation, the other environmental conditions restrict the plastics from undergoing complete biodegradation. The recovery of PET hydrolytic enzyme PETase from the bacterium *Ideonella sakaiensis* [193] and the other subsequent enzymes from marine and terrestrial metagenomes included in public databases [194] specify the omnipresence of PET degrading capacity of plastisphere in those environments. Nevertheless, the extreme oxidation via microbial biotransformation or incomplete hydrolysis of polymers leads to the generation of nano plastic which might hold the risk of ingestion through the food chain. Although the consequence of nano plastic on human health is less explored, reports [195] have suggested the possibility of translocation of microplastics (less than 150 μm) across the human gut epithelium into the lymphatic system, triggering systemic exposure and leaving an eventual effect on health after ingestion. Basiomycote *Trametes versicolor* (white-rot fungus) and *Phanerochaete chrysosporium*, Ascomycete *Humicola insolens* (soft-rot fungus), and *Engyodontium album* are the reported terrestrial fungal representatives of plastic degrader [192]. Numerous microbial species reported as potential plastic degraders are listed in (Additional file 1: Table S2). Most of the conducted plastic biodegradation experiments are in vitro, and the rate of the field degradation study has shown biodegradation rate in both marine (1.6–1.9% weight loss in 1 year) [196] and terrestrial (0.1–0.4% weight loss in 800 days) [197] environments. Various additional factors such as suboptimal degradation temperature, preference of microbes for palatable carbon sources, and polymer crystallinity play a significant role in the observed differences in the field versus laboratory-based studies. Another group of fungi, Homobasidiomycetes consisting of esterase and other polymer degrading enzymes, have been reported to degrade nylon and polyethylene in a laboratory setting [198, 199]. Though these are rare inhabitants of the marine environment, they exist in mangrove ecosystems; yet their metabolic potential is an under-explored area.

Any remediation procedure tries to eliminate the contaminant from the environment while maintaining its natural balance. Phytoremediation is an environmentally favorable approach that is commonly used [200]. Similarly, many researchers' recent discoveries [201] verified that plants accumulate microplastics. Depending on the type and level of microplastic contamination at a given site, however, several phytoremediation procedures such as phytofilteration, phytoextraction, and phytostabilization can be used. The initial point of interaction for microplastics in contaminated soil is the root zone of the plants. Li et al. (2019) [201] discovered polystyrene microbeads (0.2 m) in the root cap of lettuce plants, proving that the rhizosphere is the initial point of interaction in any phytoremediation strategy. Tolerance for MPs, accumulation proficiency, biomass output, bioaccumulation factor, root system, and other factors should all be considered when choosing plants for phytoremediation. Aside from the numerous benefits, the main downside is the potential of microplastics entering the human food chain. As a result, consideration should be given to plant selection based on MP mobilization or immobilization in the soil [202]. Plants can also collect MP pollutants from soil, according to recent results [200, 201], making them a potential phytoremediator for MP in terrestrial ecosystems. Phytoextraction (phytoaccumulation), phytostabilization, and phytofiltration are all potentially helpful phytoremediation strategies for MP-contaminated soils or water. Plant roots acquire MP pollutants and transport them to their above-ground tissues through phytoextraction or phytoaccumulation, a popular (green) remediation technique. The utilization of specific plants to immobilize MPs in the soil is the foundation of phytostabilization. The process of sorption and precipitation can help to stabilize plants. MP tends to seep into the soil's deeper layers, potentially contaminating groundwater [203]. MP are absorbed and deposited onto roots or precipitated in the rhizosphere of plants as a result of phytostabilization. This decreases or even prohibits MP mobility, reducing migration into groundwater or the atmosphere, as well as decreasing MP bioavailability, inhibiting MP distribution through the food chain. Phytofiltration is a process that uses plant roots (rhizofiltration) or seedlings (blastofiltration) to collect or adsorb MP from groundwater and aqueous waste streams rather than soil. It is comparable to phytoextraction. The rhizosphere is the soil area immediately surrounding the root surface of a plant, usually up to a few millimeters. Contaminants are absorbed or adsorbed into the root surface [204, 205]. The roots are collected and carefully disposed of after they have been wet. The site’s contamination can be reduced to an acceptable level with repeated treatments [205]

### Characterization technologies

Microplastic serves as an excellent substratum for biofilm attachment as they adsorb essential nutrients and organic matter from the surrounding environment to support microbial growth. Usually, the microbes colonize the plastics within 24 h [179] depending on several environmental factors, which significantly influence the microbial composition of the plastisphere [74]. The fluorescence microscopy (FM) and scanning electron microscopy (SEM) recognize the microbial association in the biofilm [47, 89, 92, 206, 207] (Table 3). The fluorescent self-transmissible plasmids detect the gene exchange rate between planktonic bacteria and the plastisphere community [206]. Confocal laser scanning microscopy (CLSM) properly examines the extracellular polymeric substances (EPS) of the biofilm matrix [207]. CLSM can obtain high-resolution images in various depths of a sample, generally 50–100 mm in the case of biological samples [208]. Although the previously mentioned techniques have contributed to this area of research, proper analysis and modeling of microbial colonization on plastics in different environmental settings are still needed. Variable region sequencing from the conserved 16S rRNA can detect changes in species diversity [32, 209–212]. Whole-genome sequencing (WGS) is an ideal method for observing the effect of heavy metals on resistance-related bacterial genes [213]. A combination of metagenomics and metatranscriptomics can analyze the regulation of metal resistance genes (MRGs) and antibiotic resistance genes (ARGs) at the functional/mRNA level of the whole plastisphere [32, 214]. Functional metagenomics is another convenient method to screen several resistance genes expressed in a specific environment, allowing the detection of novel functioning genes [215, 216]. Beneficial and bioremediation microorganisms were found through metagenomic analysis of sediment samples from the Ganga and Yamuna rivers [217, 218]. Furthermore, Antibiotic Resistance Genes (AMR) were discovered in the sediment metagenome of the Yamuna River [219]. The other methods of investigation are PCR [209, 210, 220], high-throughput qPCR chip technologies [212, 221], and WGS [211], although the genes investigated by the PCR technique are limited in number [64]. Metatranscriptomics for the whole transcriptome [214] and reverse transcription PCR for specific genes [222] can determine the alterations in microbial gene expression levels caused by heavy metal and plastic exposure. Although MRGs and ARGs are under investigation to study different micro-ecosystems, a comprehensive overview of the prevalence of resistance genes still needs to be done. The effect of plastics on antimicrobial resistance (AMR) at the single-cell bacterium level is possible to detect with the help of advanced technologies for the extraction and analysis of genetic elements [223]. Long-term culturing of resistant mutants exposed to subtoxic levels, followed by whole-genome sequencing (WGS), can identify the mutations during the prolonged microbial growth in a heavy metal enriched environment [224–226]. In the matter of evolution of resistant mutants accelerated before sequencing, Genome Replication Engineering Assisted Continuous Evolution is an alternative approach [226]. Two-dimensional gel electrophoresis [227], Western blotting [222], and liquid chromatography-tandem mass spectrometry (LC–MS/MS) [152] are proteomics methods frequently used in microbial monocultures. Gas chromatography mass spectrometry (GCMS) can explore the metabolites [222]. The possible impact of plastics on the proteomics and metabolomics level of the plastisphere community requires further investigation. Future next-generation sequencing (NGS) should consider the resistant gene expression levels in non-culturable bacteria present in the plastisphere. DeepARG, a tool used to explore the existing and novel ARGs and MRGs, integrated with metatranscriptomics and metagenomics, might be considered a suitable approach for this purpose [228, 229]. Although the investigation of heterogeneous modulation of gene expression of a single bacterium is possible, reports on single-cell RNA sequencing (scRNA-seq) are still scarce due to their differences from eukaryotic cells, e.g., lack of polyadenylation and low mRNA content. Microbial Split-Pool Ligation Transcriptomics (a scRNA sequencing platform), an approach adapted for Bacillus subtilis and Escherichia coli, reported several advantages, viz., (i) no requirement for single-cell physical isolation, (ii) adaptability with a broad scale of cell size and shapes, (iii) utilization of un-encapsulated and fixed cells [223, 230]. A study [231] has suggested that online separation by 1-Dimensional-LC is the most cost-effective method to amplify the number of identified proteins by MS. 1-dimensional and 2-dimensional-LCMS/MS spectrometry and mass spectrometry imaging (MSI) are promising tools to investigate the impact of microplastics on microbial proteomics and metabolomics activity levels. There are three commercially available MSI methods, which distinguish chemical compounds via their mass to charge ratio and provide chemical and spatial analysis for specific environmental samples. The chief limiting factor of a single MSI experiment is that it only yields a fraction of the metabolites present in samples [232]. Matrix-assisted laser desorption/ionization combined with FISH microscopy has linked metabolomes in complex specimens (group of 50–100 microbial cells) under investigation [233]. Microfluidics has the potential to study microbial colonization and formation of complex biofilm (Table 3) [234, 235], the impact of flow rate, and motility of bacteria on attachment surface [236]. A diverse range of resistance genes is present in both iso and heterogenic bacterial cells [237]. Droplet microfluidics might be one of the most promising technology for such investigation allowing high-throughput culturing of bacteria in a broad spectrum of isolated conditions [238, 239].

**Table 3:** Technologies to characterize the plastisphere and study the associated microbes

Type	Size	Shape	Characterization area	Tools	References
*Plastisphere substrate*					
PE,PP,PS,PVC,PET,PCL,PA	< 50 µm/1–50 µm	Fragment	Quantitative & qualitative composition of microparticles	Raman microspectroscopy	[240]
			Plastic particle-Heavy metal contamination	ICP-MS	
PET	> 300 µm	Fibre	Characterization & functional group identification of microplastics	FTIR	[64]
			Morphological & physical characterization	Stereomicroscopy	
PS,PS/PMMA blend	< 100 nm, 5–10 nm	Thin film	Chemical characterization & identification	AFM-IR	[241]
PP,PS,PVC	> 0.1 µm	Pellet, granule and powder	Identification & semi-quantification of microplastics	Py-GCToF	[242]
PE,PP,PS,PVC,PA,EPDM	0.5–0.25 mm	Fragment and fibre	Quantification of microplastics	Stereomicroscopy	[243]
			Identification of polymer composition	Py-GC/MS	
PE,PP,PS,PVC,PET,PC,PA,LDPE,HDPE	< 200 µm	Fragment	Rapid identification & quantification of microplastics	Flow cytometry	[244]
PE,OXO,AA-OXO,PHBV	9 mm	Fragment	Surface roughness & hydrophobicity	Tensiometry	[89]
PP,PS,HDPE,LDPE	4.5 mm	Fragment	Coarseness	Tensiometry	[207]
PE,PS,PP,PA,PVC	75–180 µm	Powder	Surface morphology characterization	SEM	[2]
			Crystalline composition of microplastic	XRD	
*Plastisphere microbes*					
Microbial colonization				Microfluidics	[235]
Surface covered by microbial cells & extracellular polymeric substances (EPS)				Epifluorescence microscopy	[89]
Surface colonization				AFM	
Microbial extracellular polymeric substances (EPS) measurement				CLSM	[207]
Microbial colonization				SEM	
Microbial attachment				Microfluidics	[236]
Differential gene expression of microbes under different concentrations of heavy metals				RT-PCR	[222]
Measuring changes in community structure & function				16S rRNA profiling	[32]
				Metatranscriptomics	
Quantification of ARG & mobile genetic elements (MGE)				HT-qPCR & ARG ChIP	[212]
Taxonomic & evolutionary trait analysis				WGS	[213]
Mutation in ARGs & MRGs				WGS	[225]
Evolutionary study				GREACE	[226]

*PE *Polyethylene;* PP *Polypropylene;* PS *Polystyrene;* PVC *Polyvinyl chloride;* PET *Polyethylene terephthalate;* PCL *Polycaprolactone;* PA *Polyamide;* PMMA *Poly(methyl methacrylate);* EPDM *Ethylene propylene diene monomer;* LDPE *Low-density polyethylene;* HDPE *High-density polyethylene;* OXO *additivated PE with pro-oxidant;* AA-OXO *artificially aged OXO;* PHBV *Poly(3-hydroxybutyrate-co-3-hydroxyvalerate);* PUF *Polyurethane foam;* PLA *Polylactic acid;* PU *Polyurethane; *ICP-MS *Inductively coupled plasma mass spectrometry; *FTIR* Fourier transform infrared microscopy; AFM-IR Atomic force microscope-infrared spectroscopy; *Py-GCToF* Pyrolysis–gas chromatography time of flight mass spectrometry; Py-GC/MS Pyrolysis gas chromatography mass spectrometry;  SEM Scanning electron microscopy; *XRD* X-ray diffraction; *AFM* Atomic force microscopy; *CLSM* Confocal laser scanning microscopy; *RT-PCR* Reverse transcription polymerase chain reaction; HT-qPCR High throughput quantitative real-time PCR; ARG ChIP Antibiotic resistance genes chromatin immunoprecipitation; *WGS* Whole-genome sequencing; *GREACE* Genome replication engineering assisted continuous evolution

### Significance and knowledge gaps

Considering all of these factors, variations in reported biodegradation rates are difficult to quantify, especially when the process is affected by multiple biotic and abiotic components. Biodegradation testing in laboratory settings does not adequately reflect ecological conditions, which may promote an overestimation of biodegradation levels of biodegradable plastics. As a result, biodegradation test standards should specify the limit of representativeness, and/or new particular standards should provide more alternatives for bridging the gap between natural and laboratory biodegradation. For example, depending on the geographical region, the temperature range should be adjusted. To mimic synergistic responses of biotic and abiotic variables, access to sunshine and water should also be considered. Many research has looked at the interactions between MPs and biofilms, but none have looked at how MPs physicochemical properties change as they age. More research should be done in diverse aging settings and real-world environments, taking into account the impacts of UV light, microbes, mechanical abrasion, and temperature changes. Exploring the interactions between MPs and biofilms generated by microbial colonization under ambient circumstances is extremely important and practical. These researches will give a rigorous risk assessment of the environmental impact of MPs. The amount of MP accumulated by plants from soil is still vague and unclear. As a result, more research is needed to gain a better understanding of Plant-MP-Soil interaction. Because MPs are known to absorb harmful compounds from the ambient environment, might plant collect dangerous metals alongside MP during soil uptake? This serves as the foundation for an accurate risk assessment. Studies on the potential for employing plants as phytoremediators of MP pollution in the environment are needed.

### Conclusion and future directions

As anthropogenic actions are continuously modifying our planet, the impact assessment of these activities is necessary. Plastic materials have become an integral part of our daily life and made our lives convenient. Maybe it is time to reflect on the esteem we put on such comforts. Researchers have so far established that the plastisphere microenvironment does not starkly contrast the microbial communities forming on other similarly inert substrata, primarily because biofilms forming on matured assemblages may not directly interact with the surface material. Future explorations should focus on the purpose of the plastisphere in the physical alteration and chemical biotransformation of plastic wastes, including the modification of density, size, and oxidation state of polymers. Multi-omics approaches play an indispensable part in decoding the microbial-mediated biochemical changes in plastics. Future investigations are necessary to address the many unanswered questions regarding the plastisphere research, such as the effect of chemoattractant of plastic molecules (if released) on the formation of early plastisphere. A detailed physical assessment of plastisphere (biomass, biofilm thickness, and density), generating critical data to predict the potential impact of biofilms on microplastics. The following future directions were depicted from this review work:

1. We need a profound understanding of fundamental mechanisms involved in biofilm formation, with a focus on biofilm MP interactions as every submerged surface is susceptible to microbial colonization. Experimental designs should be focused on essential parameters that influence the properties of MP. It should be determined whether these parameters vary by MP material and whether they are equivalent to naturally occurring particles of similar size. Experiments should also account for variations in physicochemical qualities as a result of weathering. These tests should be carried out in a variety of weathering environments, including UV, temperature, and mechanical wear.
2. To specify predictive models of the transport of MP particles and their associated contaminants in the aquatic environment, we need to widen the perception of biofilm-plastic hydrodynamics including vertical movement. Simulation studies on sinking and flocculation using realistic biofilm-MP complexes on micro and mesocosm sizes are required.
3. The sorption of hydrophobic organic contaminants (HOCs) to MP has gotten a lot of recognition lately. However, a logical paradigm attributing to the influence of biofilm establishment and its implications for chemical partitioning kinetics is yet to be found, which makes experimental assessments difficult. Experimental research should be supplemented by modeling studies in water-plastic-biofilm, a three-phase system.
4. Majority of experimental data on the effects of MP on biota published to date lacks the accurate composition of the test MP fragments to imitate the natural biofilm layer. MP covered by biofilms should be included from preculture incubations, and the influence of algae, fungi, and bacterial strains should be investigated. Particle controls must also mimic natural particles in terms of density, size, and biofilm formation.
5. When calculating feeding uptake and exposure effects under actual settings, the importance of biofilms for the manner and MP uptake rate by consumers should not be overlooked. The existing (ecotoxicological) research on MP intake in artificial food chains should be supplemented by differential MP uptake due to biofilm formation.
6. To measure the resilience of aquatic systems to MP contamination, we should focus on the complexity of microbial assemblages in aquatic systems and their ability to form biofilms on diverse polymer substrates (“plastisphere”). Biofilms on plastic trash should be investigated and analyzed so that we may get a functional understanding of their diversity and productivity, along with their role as vectors in disseminating microorganisms for meaningful risk evaluation.

## Supplementary Information

**Additional file 1**: **Table S1**. Global scenario of microplastic abundance on major aquatic bodies. **Table S2**. Potential microbial consortia associated with plastic biodegradation. **Fig. S1**. Common radical reactions in non-hydrolyzable polymers [269]. **A** The auto-oxidation process involves initiation by light and heat, followed by propagation and termination of a reaction which is influenced by the physical properties of the polymer. **B** Intramolecular and **C** Intermolecular hydrogen transfer reaction in polymer occurs through the abstraction and exchange of hydrogen atoms. **Fig. S2**. Polyethylene (PE), polypropylene (PP), and polyvinylchloride (PVC) are known to consist of similar carbon-carbon backbone chains. Pyrolysis (in the absence of air) is an effective depolymerization method to convert them to the respective low molecular weight aliphatic hydrocarbons [270]. According to the reports [118, 271, 272], the pyrolytic hydrocarbon products of PE are degraded through a terminal oxidation mechanism which is analogous to the n-alkane degradation pathway facilitated by microbes. **Fig. S3**. Polystyrene (PS) is broken down to its aromatic monomer styrene through pyrolysis [273] and according to O’Leary et al., (2002) [274] several microbes utilize it as a carbon source with the help of two different catabolic pathways. In the first one, which is the direct aromatic ring cleavage pathway, styrene dioxygenase (SDO) hydroxylates the aromatic ring of styrene to styrene cis-glycol. Ultimately it will generate β-D-Hydroxybutyryl-CoA by PhaB (an acetoacetyl-CoA reductase) or can be converted to PHA by PhaC (known as a PHA synthase) [275]. Another styrene metabolism pathway encompasses oxidation of its vinyl side-chain forming polyhydroxyalkanoate (PHA) as an end product.

## Data Availability

Data sharing is not applicable to this review article as no datasets were generated or analyzed during manuscript preparation.

## Code availability

Code availability is not applicable to this review article.
