# Supplementary material for: Plastisphere community assemblage of aquatic environment: plastic-microbe interaction, role in degradation and characterization technologies
Source: Environ Microbiome. 2022 Jun 24;17:32. doi: 10.1186/s40793-022-00430-4 (PMC9230103; doi:10.1186/s40793-022-00430-4)
Supplement: Supplementary file 1 — Additional file 1: Table S1. Global scenario of microplastic abundance on major aquatic bodies. Table S2. Potential microbial consortia associated with plastic biodegradation. Fig. S1. Common radical reactions in non-hydrolyzable polymers [269]. A The auto-oxidation process involves initiation by light and heat, followed by propagation and termination of a reaction which is influenced by the physical properties of the polymer. B Intramolecular and C Intermolecular hydrogen transfer reaction in polymer occurs through the abstraction and exchange of hydrogen atoms. Fig. S2. Polyethylene (PE), polypropylene (PP), and polyvinylchloride (PVC) are known to consist of similar carbon-carbon backbone chains. Pyrolysis (in the absence of air) is an effective depolymerization method to convert them to the respective low molecular weight aliphatic hydrocarbons [270]. According to the reports [118, 271, 272], the pyrolytic hydrocarbon products of PE are degraded through a terminal oxidation mechanism which is analogous to the n-alkane degradation pathway facilitated by microbes. Fig. S3. Polystyrene (PS) is broken down to its aromatic monomer styrene through pyrolysis [273] and according to O’Leary et al., (2002) [274] several microbes utilize it as a carbon source with the help of two different catabolic pathways. In the first one, which is the direct aromatic ring cleavage pathway, styrene dioxygenase (SDO) hydroxylates the aromatic ring of styrene to styrene cis-glycol. Ultimately it will generate β-D-Hydroxybutyryl-CoA by PhaB (an acetoacetyl-CoA reductase) or can be converted to PHA by PhaC (known as a PHA synthase) [275]. Another styrene metabolism pathway encompasses oxidation of its vinyl side-chain forming polyhydroxyalkanoate (PHA) as an end product. [file 40793_2022_430_MOESM1_ESM.docx]

**Table S1:** Global scenario of microplastic abundance on major aquatic bodies

| Asia | Aquatic bodies | Location | Numerical abundance of MP | Reference |
| --- | --- | --- | --- | --- |
|  | Yangtze Estuary | China | 4,137 particle m^−3^ | [245] |
|  | Lake Taihu |  | 0.3-2.5 particles m^−3^ | [246] |
|  | Vembanad Lake | India | 252.80 particles m^−2^ | [247] |
|  | Ganga river (lower stretch) |  | 99.27-409.86 particles kg^−1^ | [248] |
|  | Beijiang River | China | 0.56 particles m^−3^ | [249] |
|  | Xiangxi Bay |  | 0.11-68 particles m^−3^ |  |
| Europe | Lake Zurich | Switzerland | 0.06 particles m^−3^ | [250] |
|  | Lake Maggiore and  Grand Lac Geneva |  | 1.2 particles m^−3^ |  |
|  | Rhone River, Geneva |  | 0.29 particles m^−3^ |  |
|  | Rhone River, Chancy |  | 0.13 particles m^−3^ |  |
|  | Lake Chiusi | Italy | 2.68 to 3.36 particles m^−3^ | [251] |
|  | Lake Bolsena |  | 0.82 to 4.42 particles m^−3^ |  |
| North America | Lake Superior, Huron,  and Erie | USA | 0.27 particles m^−3^ | [252] |
|  | Illinois River |  | 1.94 to 18 particles m^−3^ | [125] |
|  | Lake Michigan |  | 17,000 particles km^−2^ | [253] |
|  | Lake Ontario | Canada | 760 particles kg^−1^ | [254] |
| Africa | South-eastern coastline | South Africa | 257.9 to 1215 particles m^−3^ | [255] |
|  | Bay of Biscay to Cape Town |  | 8.5 particles m^−3^ | [256] |
|  | Bloukrans River |  | 6.3 to 160.1 particles kg^−1^ | [257] |

**Table S2:** Potential microbial consortia associated with plastic biodegradation

| **Strain** | **Source** | **Plastic substrate** | **Reference** |
| --- | --- | --- | --- |
| *Serratia marcescens* | Ground soil | Film of LLDPE | [258] |
| *Achromobacter xylosoxidans* | Soil | Film of HDPE | [259] |
| *Zalerion maritimum* | Marine environment | Pellets of PE | [260] |
| *Oscillatoria subbrevis*  *Phormidium lucidum*; | Domestic sewage water | Film of LDPE | [261] |
| *Alcanivorax borkumensis* | Mediterranean Sea | Film of LDPE | [83] |
| *Stenotrophomonas panacihumi* | Soil of waste storage yard | Film of PP | [262] |
| *Brevibacillus agri;*  *Brevibacillus brevis*  *Brevibacillus* sp.*;*  *Aneurinibacillus aneurinilyticus;* | Landfills and sewage | Film and pellets of PP | [263] |
| *Rhodococcus* sp. *strain* 36  *Bacillus* sp. *strain* 27; | Mangrove environments | PP microplastic | [264] |
| *Acanthopleurobacter pedis;*  *Bacillus cereus;*  *Pseudomonas otitidis;*  *Bacillus aerius* | Plastic disposal sites | Film of PVC | [265] |
| *Phanerocheate chrysosporium* | Plastic disposal sites | Film of PVC | [266] |
| *Bacillus* sp. AIIW2 | Marine environment | Un-plasticized film of PVC | [267] |
| *Pseudomonas citronellolis* | Soil | Film of plasticized PVC | [268] |


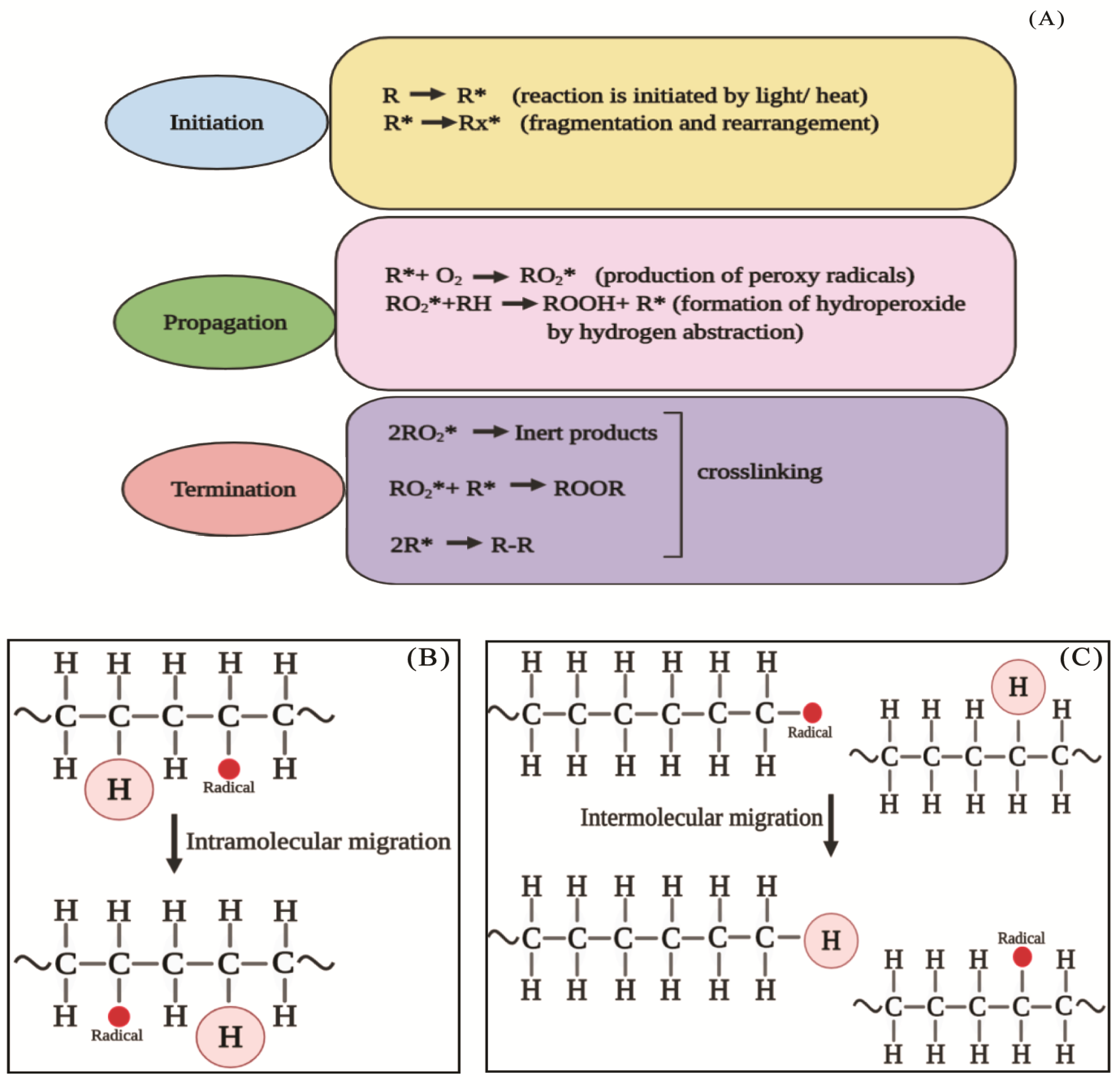


**Fig. S1.** Common radical reactions in non-hydrolyzable polymers [269]. (**A**) The auto-oxidation process involves initiation by light and heat, followed by propagation and termination of a reaction which is influenced by the physical properties of the polymer. (**B**) Intramolecular and (**C**) Intermolecular hydrogen transfer reaction in polymer occurs through the abstraction and exchange of hydrogen atoms.

**
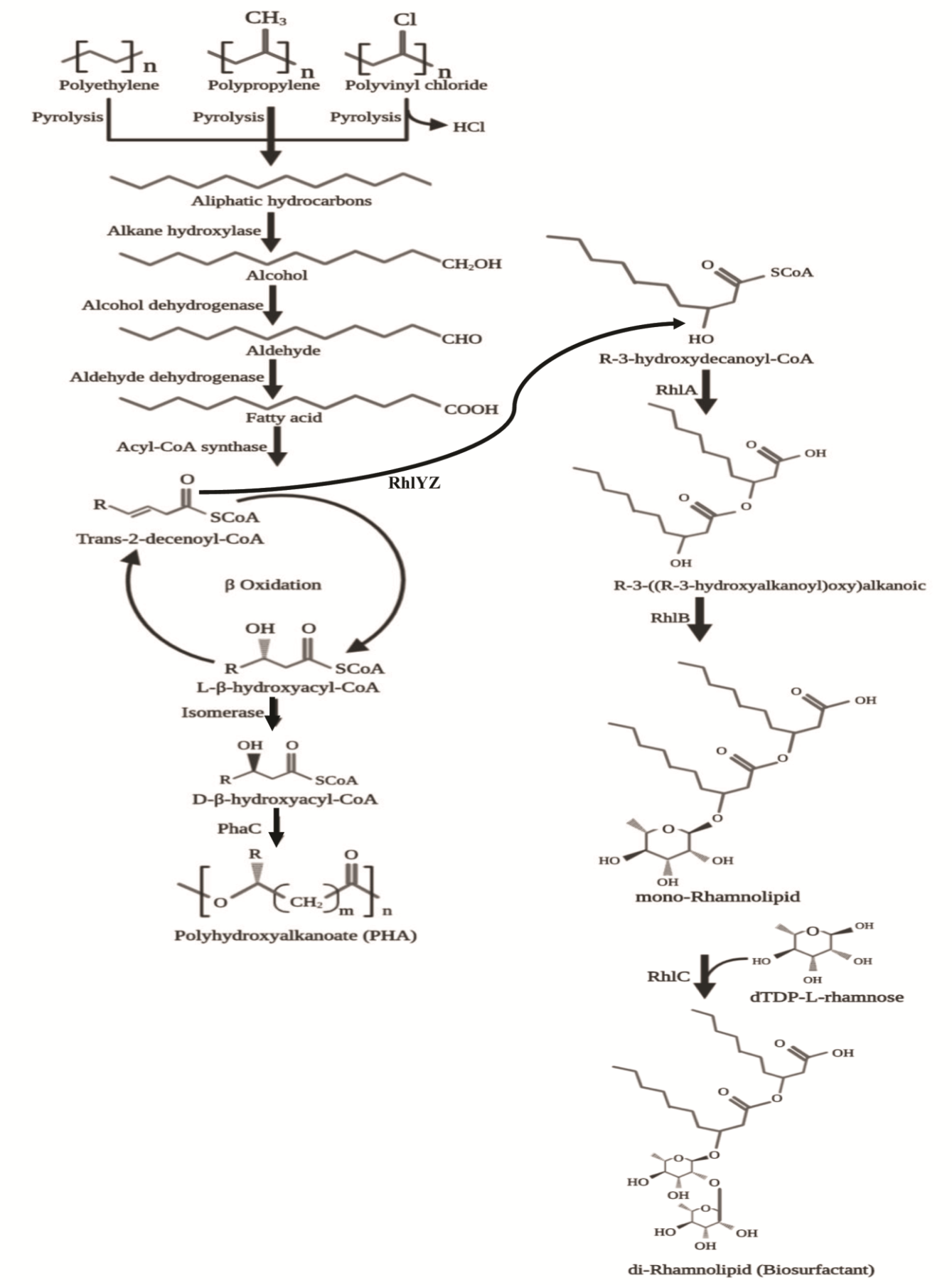
Fig. S2.** Polyethylene (PE), polypropylene (PP), and polyvinylchloride (PVC)are known to consist of similar carbon-carbon backbone chains. Pyrolysis (in the absence of air) is an effective depolymerization method to convert them to the respective low molecular weight aliphatic hydrocarbons [270]. According to the reports [118, 271-172], the pyrolytic hydrocarbon products of PE are degraded through a terminal oxidation mechanism which is analogous to the n-alkane degradation pathway facilitated by microbes.


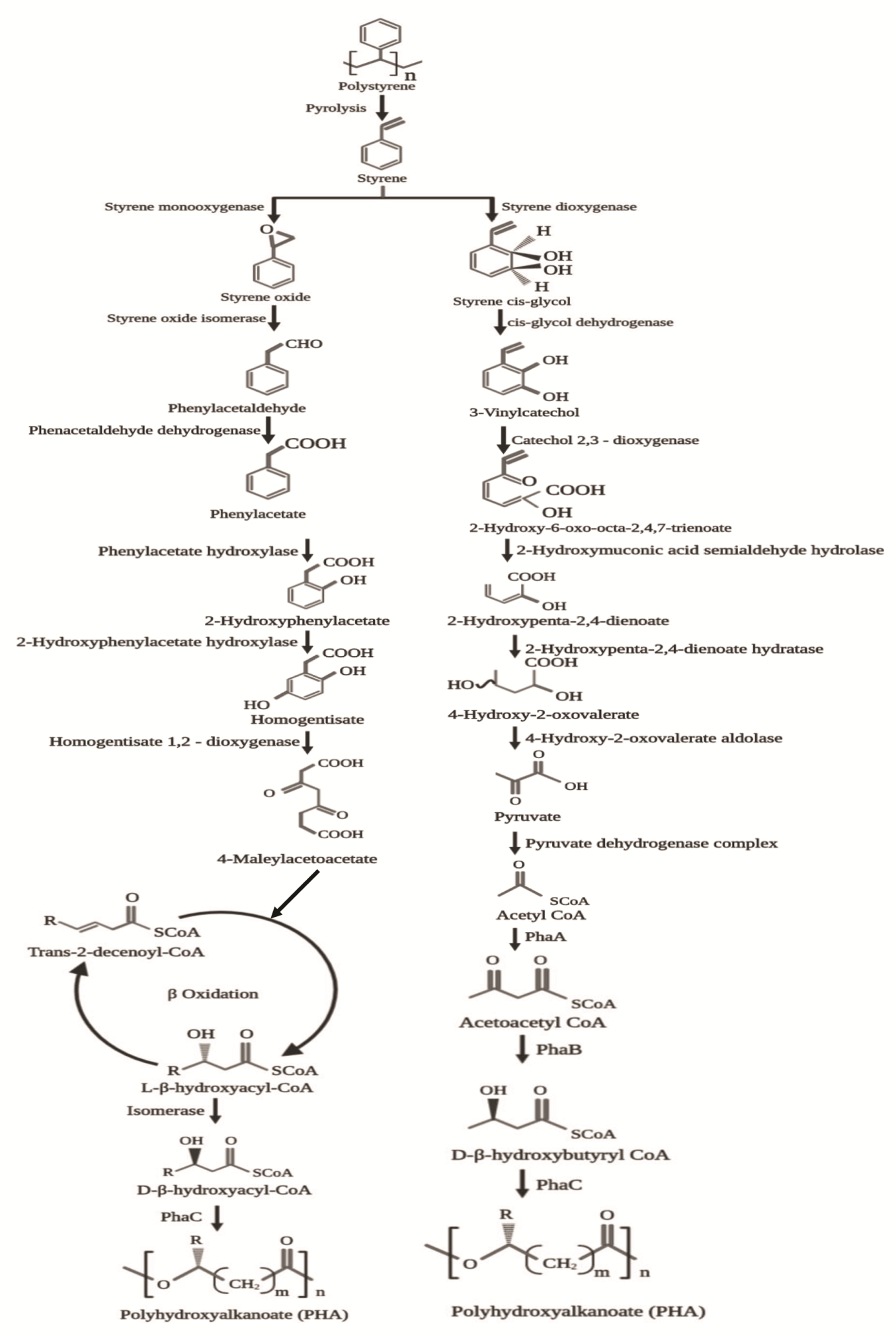


**Fig. S3.** Polystyrene (PS) is broken down to its aromatic monomer styrene through pyrolysis [273] and according to O’Leary et al., (2002) [274] several microbes utilize it as a carbon source with the help of two different catabolic pathways. In the first one, which is the direct aromatic ring cleavage pathway, styrene dioxygenase (SDO) hydroxylates the aromatic ring of styrene to styrene cis-glycol. Ultimately it will generate β-D-Hydroxybutyryl-CoA by PhaB (an acetoacetyl-CoA reductase) or can be converted to PHA by PhaC (known as a PHA synthase) [275]. Another styrene metabolism pathway encompasses oxidation of its vinyl side-chain forming polyhydroxyalkanoate (PHA) as an end product.
